# Retraction Note: In vitro antioxidant, anticancer, anti-inflammatory, anti-diabetic and anti-Alzheimer potentials of innovative macroalgae bio-capped silver nanoparticles

**DOI:** 10.1007/s11356-024-34308-4

**Published:** 2024-07-09

**Authors:** Manal N. Abdel Azeem, Osama M. Ahmed, Mohamed Shaban, Khaled N. M. Elsayed

**Affiliations:** 1 Physiology Division, Zoology Department, Faculty of Science, Beni-Suef University, Beni-Suef, Egypt; 2 Nanophotonics and Applications (NPA) Lab, Physics Department, Faculty of Science, Beni-Suef University, Beni-Suef, 62514 Egypt; 3 Department of Physics, Faculty of Science, Islamic University in Almadinah Almonawara, 42351 Almadinah Almonawara, Saudi Arabia; 4 Botany and Microbiology Department, Faculty of Science, Beni-Suef University, Beni-Suef, Egypt

**Retraction Note: Environmental Science and Pollution Research (2022) 29:59930-59947**

10.1007/s11356-022-20039-x

The Publisher has retracted this article in agreement with the Editor-in-Chief. An investigation by the publisher found a number of articles, including this one, with a number of concerns, including but not limited to compromised peer review process, inappropriate or irrelevant references, containing nonstandard phrases or not being in scope of the journal. Based on the investigation's findings the publisher, in consultation with the Editor-in-Chief therefore no longer has confidence in the results and conclusions of this article.

The authors have not responded to correspondence regarding this retraction.

